# Influence of Preparation Methods of Chitooligosaccharides on Their Physicochemical Properties and Their Anti-Inflammatory Effects in Mice and in RAW264.7 Macrophages

**DOI:** 10.3390/md16110430

**Published:** 2018-11-02

**Authors:** Ángela Sánchez, María Mengíbar, Margarita Fernández, Susana Alemany, Angeles Heras, Niuris Acosta

**Affiliations:** 1 Instituto de Estudios Biofuncionales, Departamento de Química en Ciencias Farmacéuticas, Facultad de Farmacia, Universidad Complutense de Madrid, 28040 Madrid, Spain; assanchez@iib.uam.es (Á.S.); marianmengibar@hotmail.es (M.M.); 2 Instituto de Investigaciones Biomédicas “Alberto Sols”, Consejo Superior de Investigaciones Científicas & Universidad Autónoma de Madrid, (CSIC-UAM), 28029 Madrid, Spain; marfermar47@gmail.com (M.F.); salemany@iib.uam.es (S.A.)

**Keywords:** chitooligosaccharides, anti-inflammatory action, RAW264.7 macrophage

## Abstract

The methods to obtain chitooligosaccharides are tightly related to the physicochemical properties of the end products. Knowledge of these physicochemical characteristics is crucial to describing the biological functions of chitooligosaccharides. Chitooligosaccharides were prepared either in a single-step enzymatic hydrolysis using chitosanase, or in a two-step chemical-enzymatic hydrolysis. The hydrolyzed products obtained in the single-step preparation were composed mainly of 42% fully deacetylated oligomers plus 54% monoacetylated oligomers, and they attenuated the inflammation in lipopolysaccharide-induced mice and in RAW264.7 macrophages. However, chitooligosaccharides from the two-step preparation were composed of 50% fully deacetylated oligomers plus 27% monoacetylated oligomers and, conversely, they promoted the inflammatory response in both in vivo and in vitro models. Similar proportions of monoacetylated and deacetylated oligomers is necessary for the mixtures of chitooligosaccharides to achieve anti-inflammatory effects, and it directly depends on the preparation method to which chitosan was submitted.

## 1. Introduction

Inflammation is a response of the organism to injury, and a prolonged or intense inflammatory event can lead to the development of several diseases. Different cell types and inflammatory mediators such as cytokines act in coordination to resolve the inflammatory event successfully [1]. Lipopolysaccharide (LPS) is a common pathogen-associated molecular-pattern contained in large amounts in the cell walls of Gram-negative bacteria, and stimulates cells of the innate immune system by its ligation to Toll-like receptor 4 (TLR4) [2]. Different cell types, such as neutrophils and macrophages, respond to LPS by releasing potent inflammatory mediators [3,4]. Neutrophils are typically the first leukocytes to be recruited to the inflammatory site and are capable of eliminating pathogens by multiple mechanisms [5,6]. Macrophages are also inflammatory cells with the capacity to acquire a variety of different functional phenotypes in response to the extracellular signals [7,8].

Mitogen-activated protein kinases (MAPKs) are highly conserved families of serine/threonine protein kinases involved in a variety of fundamental cellular processes which play a key role in signal transduction [9,10]. These MAPK signaling pathways induce a secondary response by increasing the expression of inflammatory cytokines and chemokines. The binding of LPS to TLR4 engages multiple signal transduction pathways, including three groups of MAPK: extracellular-signal-regulated kinases (ERKs); the cJun NH_2_-terminal kinases (JNKs); and the p38 MAP kinases [11]. The importance of the different MAPKs in controlling cellular responses to the environment by regulating gene expression has made them a priority for research related to many human diseases [9]. Indeed, the ERK, JNK, and p38 pathways are all molecular targets for drug development [12].

Chitosan, the main derivative of chitin, is the essential polymer to produce chitooligosaccharides (COSs), which are linear co-oligomers of *N*-acetyl glucosamine (GlcNAc) and glucosamine (GlcN) units in varying proportions. COSs have been reported to exhibit numerous biological functions, such as antibacterial, anti-inflammatory, antioxidant, immunity-enhancing, antitumor, etc. [13,14,15,16]. The biological effects of COSs depend on their degree of polymerization (DP) or average molecular weight (Mw), the degree of *N*-acetylation (DA), and/or the pattern of *N*-acetylation (PA), which in turn depends on the source of chitosan and, most importantly, on the hydrolysis strategy [17]. The relation between the biological function and the structure of COSs is still not well understood [18], making it difficult to establish a hypothesis about their structure–function relationship. The bioactivities of COSs are often tested using relatively poorly characterized chitooligomers, so it is essential to use well-defined and highly purified COS preparations with defined physicochemical characteristics to understand their bioactivity [13].

Currently, COSs are produced by different physical and chemical methods [19,20,21,22], but enzymatic procedures present significant advantages, especially when COSs will be used for biomedical applications [23,24,25]. Partial enzymatic hydrolysis of chitosan with specific enzymes such as chitosanases has been proposed as an alternative to the other hydrolysis methods [26] because the resulting COS can be defined [27].

The focus of this study was to clarify the relationship between the preparation methods of COSs, their physicochemical structure, and their effects related to (anti)-inflammatory properties. Our group optimized the reproducibility of COS preparation methods, and we prepared several COSs by enzymatic depolymerization, with and without previous chemical degradation of chitosan. The structure and composition of chitooligosaccharides were studied in detail and the roles of the anti-inflammatory properties of these COSs using in vitro and in vivo models were evaluated.

## 2. Results

### 2.1. Structural and Physicochemical Characterization of COSs

**Figure 1:**
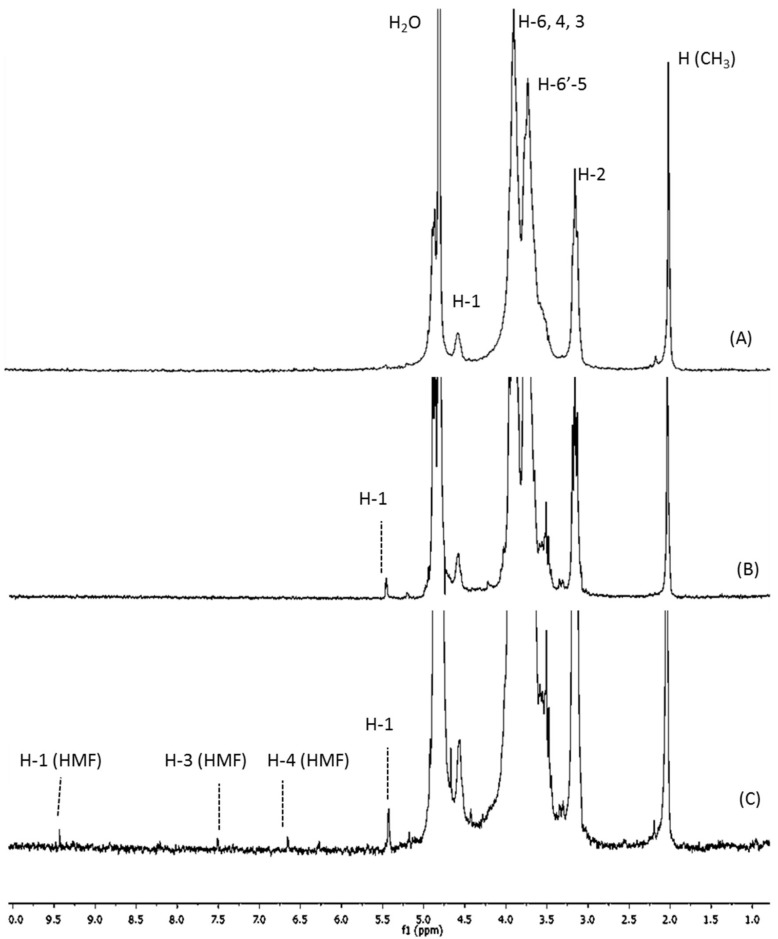
^1^H-NMR spectra. (**A**) Native chitosan; (**B**) P1COS; (**C**) P2COS. (**B**,**C**) show the signal of H1 corresponding to deacetylated reducing ends. (**C**): The assignments of 5-hydroxymethyl-2-furfural (HMF) were H-1 (9.43 ppm), H-3 (7.51 ppm), and H-4 (6.66 ppm) [29].

**Figure 2:**
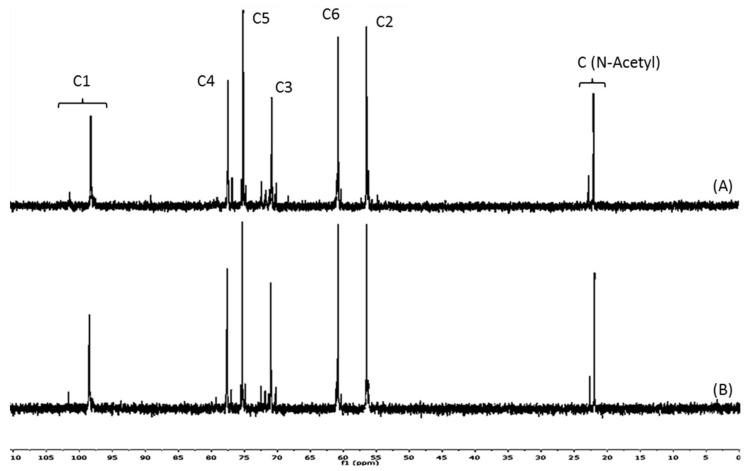
^13^C-NMR spectra. (**A**) P1COS obtained in the one-step enzymatic method; (**B**) P2COS obtained in the two-step chemical-enzymatic method.

The chitosanolytic activity was checked by HP-SEC, and COSs with lower Mw than initial chitosan were successfully obtained by both methods (Table 1). Low molecular weight chitosan (LMWC) with 32 kDa was obtained in the first step of the two-step process (P2), which led to P2COS (Mw 5 kDa), with lower Mw than P1COS (Mw 8 kDa). In the ^1^H-NMR spectra, the proton signals (H1–H6) showed the preservation of chitosan structure in both P1COS and P2COS (Figure 1). A slight signal assigned to 5-hydroxymethyl-2-furfural (HMF) was observed in P2COS (Figure 1C). Using ^13^C-NMR, it was checked that the internal structures of P1COS and P2COS were not altered in their preparation processes (Figure 2). The pattern of *N*-acetylation was 0.8 and 0.9 respectively for P1COS and P2COS, corresponding to a random pattern of acetylated monomers distribution [28].

**Figure 3:**
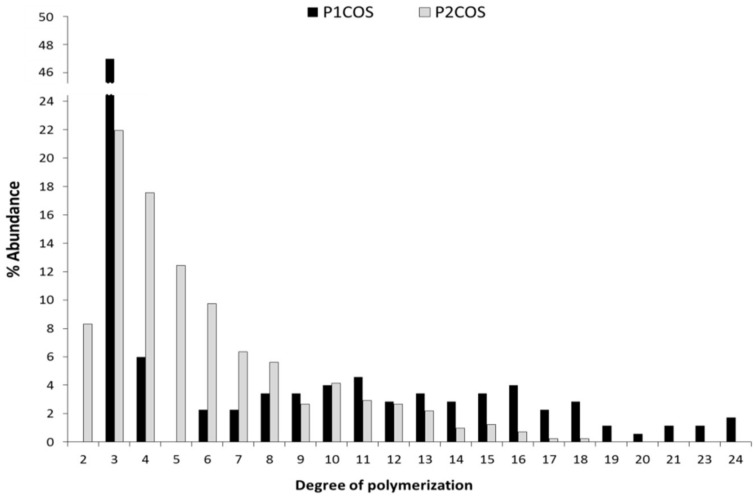
The abundance of the percentage of the degree of polymerization present in both mixtures of P1COS and P2COS.

Both P1COS and P2COS were analyzed by MALDI-TOF-MS to determinate their composition and abundance of the degree of polymerization (Table 1 and Figure 3). COSs showed the typical profile of molecular composition for chitooligosaccharides, and their DP ranged from 3 to 24 in P1COS and from 3 to 17 in P2COS. This result is in concordance with the lower Mw determined previously for this fraction by HP-SEC. Both COSs were composed of fully deacetylated oligomers (A_0_) and mono-(A_1_), di-(A_2_), and tri-acetylated (A_3_) oligomers. Larger acetylated oligomers were not detected (Table 1).

### 2.2. Role of P1COS and P2COS in the Peritoneal Migration of Neutrophils

**Figure 4:**
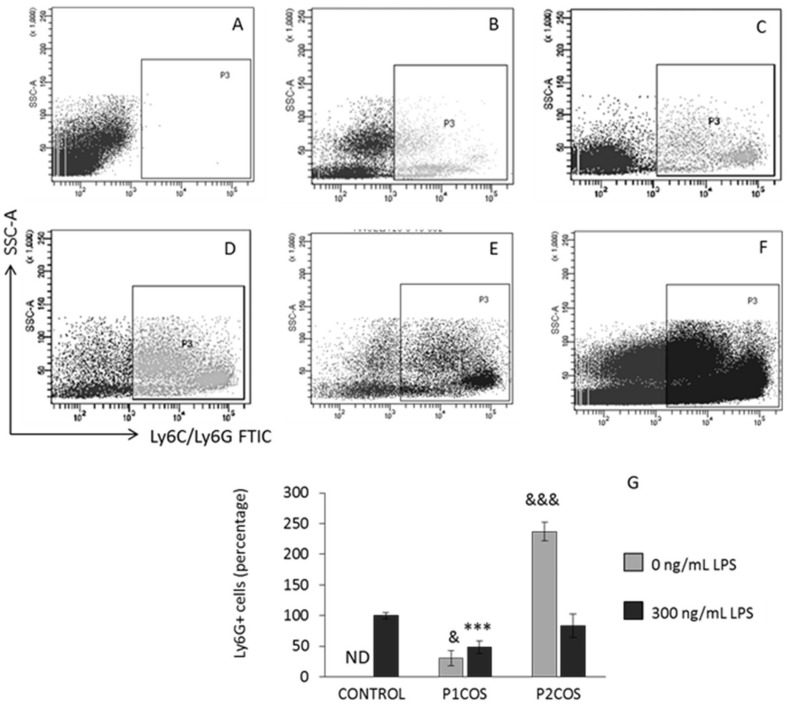
Neutrophils population from mice treated with lipopolysaccharide (LPS), P1COS, P2COS, LPS plus P1COS, and LPS plus P2COS. Mice received an intraperitoneal injection of LPS (1 mg/kg) or of either P1COS or P2COS (10 mg/kg) alone or together with LPS, and 24 h later the levels of circulating neutrophils Ly6G+ cells were analyzed by flow cytometry. (**A**–**F**) Representative flow cytometry graphs showing the percentage of circulating Ly6G+ cells. (**A**) Control group; (**B**) P1COS; (**C**) P2COS; (**D**) LPS; (**E**) LPS and P1COS; (**F**) LPS and P2COS. (**G**) The means ± SD (*n* = 6) of the frequency of cells relative to the total number of circulating cells. Asterisks represent the significance with respect to the control LPS-induced mice and “&” with respect to the control without LPS. The 100% inflammation corresponds to 2.1 × 10^5^ ± 2.5 × 10^4^ Ly6G+ cells. ND: not detectable.

Intraperitoneal injection of LPS in mice induces the accumulation of neutrophils in the peritoneum [30]. To test the implication of P1COS and P2COS in anti-inflammatory response in vivo, we injected mice with LPS, P1COS, and P2COS, as well as LPS together with P1COS or P2COS. Twenty-four hours later, the neutrophil population recruitment to the peritoneum was determined by flow cytometry (Figure 4). P1COS promoted a decrease in the neutrophil population in LPS-induced mice, meaning that P1COS was able to attenuate the acute inflammatory response. Conversely, P2COS generated the most elevated neutrophil recruitment to the peritoneum, even higher than LPS, indicating a pro-inflammatory behavior for this COS. To further analyze the role of P1COS and P2COS in inflammation, we next decided to study how they modulate the LPS response in vitro using RAW264.7 macrophage.

### 2.3. Role of P1COS and P2COS on LPS-Activated RAW264.7 Macrophage Cells

**Figure 5:**
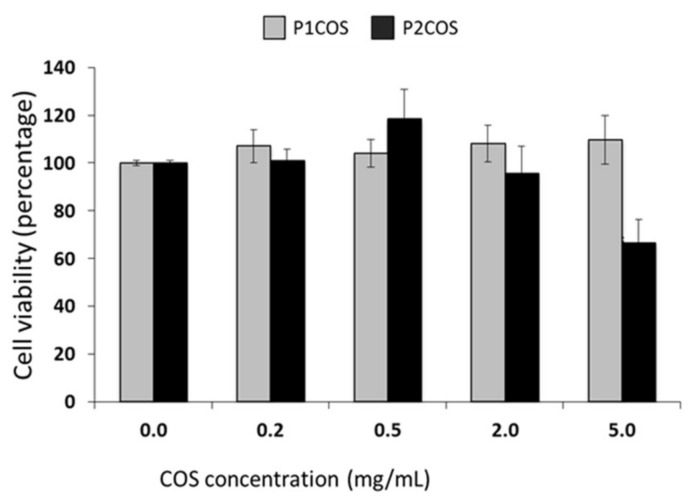
Effects of P1COS and P2COS on the viability of RAW264.7 cells. Cells were treated for 4 h with P1COS or P2COS (0–5 mg/mL), and relative cell viability was measured. The panel shows the mean ± SD from three independent experiments relative to the total cell viability in non-treated cells. Asterisk represents the significance with respect to the non-treated control.

First, we analyzed the cytotoxicity of P1COS and P2COS on RAW264.7 cells by assaying the viability of the cells incubated with different concentrations of both compounds. P1COS did not affect the viability of the cells at any of the concentrations tested (Figure 5). On the contrary, P2COS affected the viability of macrophage at 5 mg/mL, decreasing the cell survival about 60%. Therefore, 2 mg/mL of P2COS was used for in vitro experiments.

**Figure 6:**
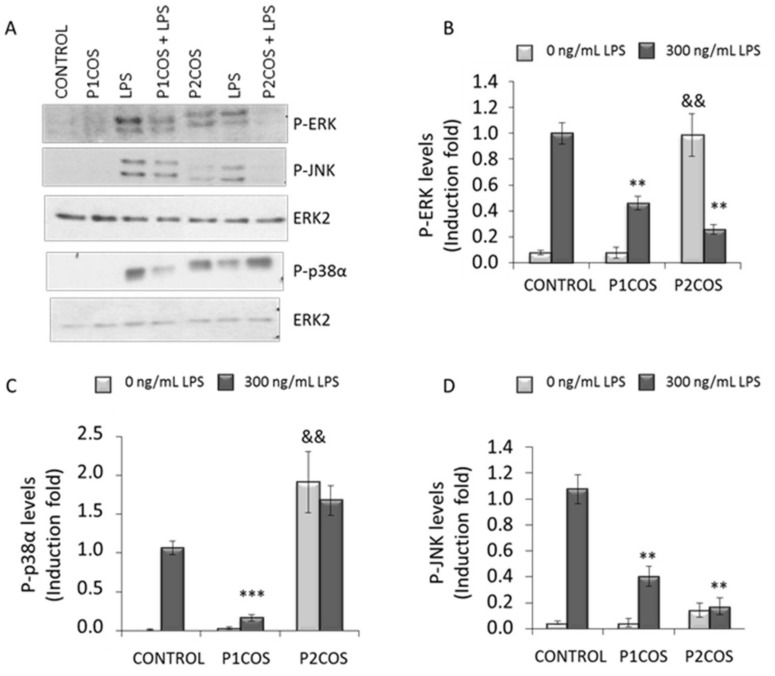
Extracellular-signal-regulated kinase (ERK), cJun NH_2_-terminal kinase (JNK), and p38α phosphorylation levels in RAW264.7 cells treated with P1COS or P2COS with or without LPS. Cells were stimulated with LPS (300 ng/mL) or either P1COS or P2COS (2 mg/mL) with or without LPS for 30 min. P-ERK, P-JNK, and P-p38α were analyzed by Western blots, and the total protein ERK2 was analyzed as loading controls. (**A**) Representative Western blots of all experimental conditions. (**B**–**D**) The graphs show the means ± SD (*n* = 3) of protein levels fold induction relative to the total of phosphorylated protein in LPS-induced cells, after normalizing values. Asterisk represents the significance with respect to the control LPS-induced cells and “&&” with respect to the control without LPS.

Subsequently, RAW264.7 macrophage cells were treated with P1COS or P2COS, with or without LPS, to assess the effect on the activation state of ERK, JNK, and p38α by Western blotting. The activation levels of these kinases were evaluated by determining their phosphorylation state [9]. Both P1COS and P2COS significantly attenuated the activation of ERK and JNK in LPS-induced macrophage (Figure 6B,D). On the other hand, p38α was attenuated by P1COS but not by P2COS, which promoted a strong increase in its activation levels (Figure 6C). Interestingly, stimulation of cells with P2COS increased the phosphorylation of both p38α and ERK. Indeed p38α phosphorylation was even higher when it was stimulated with P2COS rather than with LPS. However, P1COS did not activate any of the MAP kinases in this cell system.

**Figure 7:**
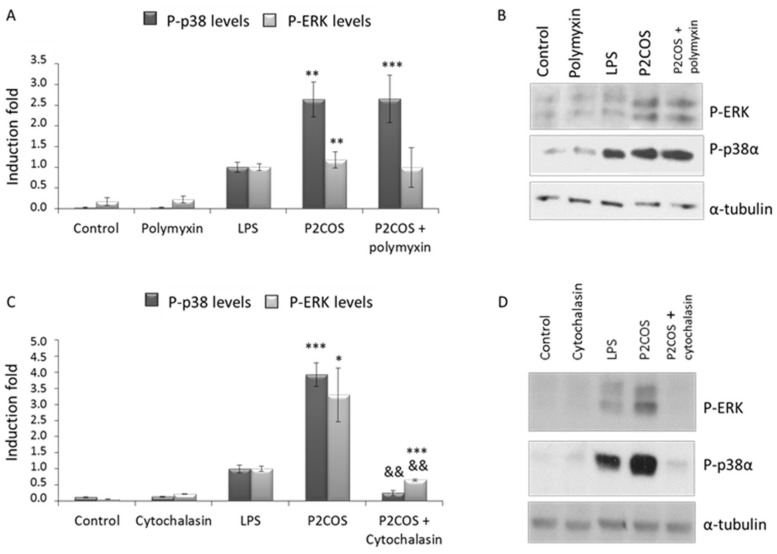
Protein levels of P-ERK and P-p38α in RAW264.7 after P2COS treated (2 mg/mL) for 30 min. Cells were pre-incubated or not with polymyxin-B (1 µg/mL) or cytochalasin-B (10 µg/mL) for 30 min before stimulation with COS, after which the levels of P-ERK and P-p38 were determined by Western blot analysis. As a loading control, membranes were blotted with anti-α-tubulin. (**B**,**D**) Representative Western blots of all experimental conditions. (**A**,**C**) The graphs show the means ± SD (*n* = 3) of protein levels fold induction relative to the total phosphorylated protein in LPS-induced cells, after normalizing values. Asterisk represents the significance with respect to the non-treated cells and “&&” with respect to the P2COS plus polymyxin-B or cytochalasin-B respectively.

To investigate whether P2COS activates RAW264.7 in the same way as LPS, cells were pre-incubated with two different compounds: polymyxin-B, known to inhibit TLR4 activation [31], and cytochalasin-B, as an actin cytoskeleton disruptor [32], and then stimulated with P2COS. The presence of polymyxin-B did not affect the activation of p38α and ERK promoted by P2COS in RAW264.7 cells (Figure 7A,B). However, the presence of cytochalasin-B was able to partially inhibit the effect of P2COS, drastically decreasing activation levels of ERK and p38α (Figure 7C,D).

## 3. Discussion

### 3.1. The Relation between P1COS and P2COS Preparation Methods and Their Composition

Due to the action of the chitosanase, which specifically breaks glycosidic bonds GlcN–GlcN or GlcN–GlcNAc, higher-intensity signals corresponding to the new deacetylated reducing ends generated (5.4 ppm, assigned to H-1 of GlcN) were detected in P1COS and P2COS [26,33], and a slight increase of this signal was seen in P2COS. In the acidic hydrolysis step of P2, major unstable 2,5-anhydro-d-mannose reducing ends would be produced, leading to the generation of Schiff bases that in turn contribute to HMF formation [29]. The intermediate products of Maillard reactions are advanced glycation end products (AGEs) that emit fluorescence [34], but we found a negligible signal in P2COS. The random pattern of acetylation determined for P1COS and P2COS suggests that the preparation methods did not affect the internal structure of COSs.

As in the chitosanolytic hydrolysis, the GlcN–GlcN or GlcN–GlcNAc glycosidic bonds are attacked by the nitrous acid [29], decreasing the substrate viscosity. Therefore, the double hydrolysis of P2 facilitates the production of COS with lower Mw. The molecular composition was described as D_n-m_A_m_, where m has values between 0 and 3 and n has values between the minimum and the maximum DP [23,35]. P2COS had a slightly higher percentage of fully deacetylated sequences (A0 = 50%) than P1COS (A0 = 42%), in concordance with results obtained by ^1^H-NMR. Conversely, monoacetylated oligomers (A1 = 54%) were dominant in P1COS and more abundant than in P2COS (A1 = 27%). Therefore, COSs with a higher percentage of A0 oligomers and lower Mws were generated in P2 as we previously described for chitosan from another source [36].

### 3.2. Role of P1COS and P2COS in LPS-Induced Mice and LPS-Activated RAW264.7 Macrophage Cells

It has been reported that the Mw and DP distribution of COSs influence their anti-inflammatory capacity [37,38] and that this is dose-dependent [39], showing a better anti-inflammatory activity for COS with Mw lower than 10 kDa. It has been proposed that the anti-inflammatory effects of COS in LPS-induced mice could be due to the formation of a stable water-soluble COS–LPS complex through electrostatic interactions [40], making the interaction between LPS and its receptor TLR4 [41] difficult and promoting a partial inhibition of its effect. The interaction of COS–LPS significantly depends on the LPS structure, concentration, COS Mw, and the parameters of the medium where the complexing occurs [42]. Hydroxyl groups of COS would be donors of a proton to form hydrogen bonds, and their *N*-acetyl groups would be collaborating in the hydrophobic interaction that together would stabilize the complex [43,44].

The protein ERK is involved in the regulation of main functions in differentiated cells (e.g., mitosis or apoptosis), and many different stimuli activate the ERK pathway [9,45,46,47]. JNK and p38α MAPK, also called stress-activated protein kinases, have been implicated in a variety of cellular processes, including cell proliferation, differentiation, and death [48]. The natural peptide polymyxin-B is a potent antibiotic that binds to LPS and neutralizes it [31]. P2COS was able to activate inflammation in a TLR4-independent way since ERK and p38α were activated in the presence of polymyxin-B (Figure 7A,B). However, the inflammation promoted by P2COS was significantly attenuated in the presence of cytochalasin-B since the activation levels of P-ERK1 and P-p38α decreased drastically (Figure 7C,D), meaning that this P2COS needs to be internalized by the macrophage to promote inflammation. The attenuation of p38α was stronger than ERK, which remained active. Therefore, it seems that the activation promoted by P2COS is not exclusively by phagocytosis, since significant levels of P-ERK were detected.

Both P1COS and P2COS showed similar DA (Table 1), but P1COS contained a higher proportion of fully deacetylated (A0) and monoacetylated (A1) oligomers (42% and 54%, respectively) than P2COS, which was composed of 50% of A0 and 27% of A1 oligomers. The higher proportion of A0 and A1 oligomers of P1COS could be crucial to establishing a stable complex with the LPS. This composition would contribute to stabilizing the LPS–COS complex. P1COS prepared by the enzymatic single-step method seems to be an optimal option to produce COSs with anti-inflammatory activity, and the different composition of P2COS would not promote the LPS–COS complex formation, making it difficult to attenuate the inflammatory response.

The composition of P2COS probably allows it to complex with LPS as described above, promoting the attenuation of MAPK in LPS-induced RAW264.7 cells (Figure 6). However, preparation method P2 would influence its pro-inflammatory behavior, promoting the activation of ERK and p38 MAPK, and resulting in P2COS being recognized by cells as an alarm signal. Compared to P1COS, the higher presence of deacetylated reducing ends would also contribute to pro-inflammatory effects. The HMF detected, a common product of Maillard reactions and generated by heat in some food [49], showed no evidence of toxicity [50]. However, the participation of this compound in more advanced stages of Maillard reactions cannot be discarded [51,52]. This fact could contribute to the pro-inflammatory effects shown for P2COS in mice and in the RAW264.7 macrophage. The AGEs are produced in the cell as a consequence of carbohydrate and protein degradation promoted by cellular aging. The negligible presence of AGEs that are closely related to cardiovascular diseases [34] could contribute to the pro-inflammatory response of P2COS. Many intracellular receptors recognize different exogenous molecules [53]. Macrophages have receptors for AGEs (RAGEs) [54] through which they activate the ERK pathways between others [55]. These RAGEs could recognize part of P2COS, but more experiments would be needed to investigate this interaction. Based upon this observation, it seems that the preparation of COSs by a single-step process based on an enzymatic hydrolysis of chitosan is a better option for the generation of COSs with anti-inflammatory properties.

## 4. Materials and Methods

### 4.1. Preparation of COSs

Two kinds of COS were prepared from chitosan (89 kDa; DA 17%) obtained from fresh North Atlantic shrimp shells *Pandalus borealis* (Novamatrix, Sandvika, Norway). This chitosan (0.5%, *w*/*w* in 0.5 M acetic acid) was filtered through a series of glass Buchner funnels (250 to 16 µm), precipitated with 10% (*w*/*v*) NaOH aqueous solution, and washed to neutrality with distilled water before its use. One Mw range fraction of COS (Mw 5–10 kDa) was enzymatically prepared using chitosanase EC 3.2.1.132 from *Streptomyces griseus* (Sigma-Aldrich, St. Louis, MO, USA) in the single-step process (P1). The COS was named P1COS, was dialyzed against distilled water until complete salt elimination, and freeze-dried. The other method to prepare COS was performed in two steps (P2). In the first stage, chitosan was depolymerized with nitrous acid [56] to obtain a low molecular weight chitosan (LMWC), which was enzymatically degraded in the second stage with chitosanase EC 3.2.1.132. One Mw range fraction of COS (Mw 5–10 kDa) was isolated, dialyzed, and freeze-dried, and it was named P2COS. All details of preparation methods were previously reported [36]. Prior to stimulation of the anti-inflammatory effect, an exhaustive physicochemical characterization of COS was performed. Both processes were monitored by HP-SEC, and a standard chitosan curve was used to determine the Mw of LMWC, P1COS, and P2COS [57].

### 4.2. Structural and Physicochemical Characterization of COS

^1^H-NMR spectra were obtained on a 300 MHz Bruker Advance Spectrometer (Bruker Ettinglen, Bremen, Germany). Chitooligosaccharides were dissolved (0.5%, *w*/*v*) in 1% DCl/D_2_O (Sigma-Aldrich), measurements were recorded at 25 °C, the acquisition time was 1 s with 32 scans, and DA was determined according to [58]. ^13^C-NMR spectra were obtained on an Agilent DD2 600 Spectrometer (Agilent Technologies, Santa Clara, CA, USA). Samples were prepared as described above. The acquisition time was 1.5 s with 5788 scans and 600 MHz. The PA was determined as described in [28]. MALDI-TOF-MS was performed in a Bruker Ultraflex MALDI-TOF mass spectrometer (Bruker-Daltonik, Bremen, Germany) in positive ion mode as detailed by Madhuprakash, El Gueddari, Moerschbacher, and Podile [59].

### 4.3. Animal Model

Wild type C57BL/6 mice between the ages of 10–12 weeks were used for in vivo experiments. The animals were handled in accordance with Institutional Guidelines for the Care and Use of Laboratory Animals in research, the relevant European Council Directive (2010/63/EU), and Spanish law (R.D.1201/2005), with the approval of the Ethics Committee of the Consejo Superior de Investigaciones Científicas. Mice were intraperitoneally injected with LPS (*Salmonella enterica* serotype Typhimurium, Sigma-Aldrich, 1 mg/kg) alone or together with P1COS or P2COS (10 mg/kg), which were previously dissolved in PBS and sterilized by filtration through a 0.2 µm filter. After 24 h, peritoneal cells were isolated and stained with Anti-Gr1 (Ly6C/Ly6G) FITC (Bioscience, Madrid, Spain). The neutrophil population was determined by flow cytometry (FC 500 MPL, Beckman-Coulter, Indianapolis, IN, USA).

### 4.4. Cell Culture, Stimuli, and Western Blotting Analysis

Murine RAW264.7 macrophage was cultured in Dulbecco’s modified Eagle’s medium (DMEM) supplemented with 10% heat-inactivated fetal bovine serum (FBS), 25 mM HEPES, 100 U/mL penicillin, 0,5 µg/mL streptomycin, and 2 mM glutamine. Cells were cultured at 37 °C in a humidified atmosphere (5% CO_2_). For in vitro studies, COSs were prepared in culture medium and sterilized by filtration. Prior to stimulation with P1COS or P2COS, non-toxic concentrations of COSs were selected after evaluating their cytotoxicity in the range 0.2 to 5 mg/mL for 4 h [60] by 3-(4,5-dimethylthiazol-2-yl)-2,5-diphenyltetrazolium bromide (MTT) test. For experiments, raw cells were cultured overnight in DMEM with 0.1% FBS. Subsequently, cells were treated with 2 mg/mL of P1COS or P2COS with or without LPS (300 ng/mL) for 30 min. After that, cells were collected and protein extracts were generated and subjected to Western blot analysis [61] using anti-phospho-Erk1/2 (P-ERK, P-p44/42MAPK, Cell-Signaling, Leiden, The Netherlands), anti-phospho-p38α (P-p38, P-T180/Y182, Cell Signaling, Leiden, The Netherlands), and anti-phospho-Jnk1/2 (P-JNK, P-T183/Y185 JNK1/2, Promega-Biotech, Madrid, Spain) antibodies. In some experiments, cells were pre-treated with 1 µg/mL of polymyxin-B (Sigma-Aldrich, St. Louis, MO, USA) or 10 µg/mL of cytochalasin-B (*Drechslera dematioidea*, Sigma-Aldrich, St. Louis, MO, USA) for 30 min before COS treatment.

### 4.5. Statistical Analysis

Statistical significance was determined by applying student’s *t*-test using GraphPad-Prism software. Results shown are mean ± standard error. *p*-values are represented in the figures with asterisks: * *p* < 0.05, ** *p* < 0.01, and *** *p* < 0.001.

## 5. Conclusions

P1COS prepared by the single-step enzymatic method was able to attenuate the inflammatory response in LPS-induced mice and in LPS-activated murine RAW264.7 macrophage cells. Preparation of COS by this method generates oligomers with a lower proportion of fully deacetylated oligomers compared to our proposed two-step methods. Moreover, its balance with acetylated oligomers seems to be a crucial requirement for using P1COSs as anti-inflammatory agents. On the contrary, the high proportion of fully deacetylated reducing ends and the lower percentage of acetylated oligomers of P2COS could contribute to destabilizing the LPS–COS complex, decreasing its anti-inflammatory effect. In addition, the intermediate products of the Maillard reactions of P2COS could contribute to its pro-inflammatory action in vivo and in vitro. The development of detailed methods to prepare COSs and to study their physicochemical structure is essential to establishing a relationship with their biological function. The use of COSs as anti-inflammatory agents in biotechnological applications will depend on a good knowledge of the structure–function relation.

## Figures and Tables

**Table 1:** Average molecular weight (Mw), degree of *N*-acetylation (DA), zeta potential (ζ), pattern of *N*-acetylation (PA), and percentage of acetylated oligomers. P1COS: chitooligosaccharide (COS) prepared using the one-step method; P2COS; COS prepared using the two-step method.

Sample	Mw (kDa)	DA (%)	ζ (mV)	PA	A_0_ (%)	A_1_ (%)	A_2_ (%)	A_3_ (%)	A_4_–A_24_ (%)
P1COS	8	13	56.4 ± 4.9	0.8	42	54	3	1	0
P2COS	5	11	33.1 ± 1.9	0.9	50	27	14	9	0

(A_0_), mono- (A_1_), di- (A_2_), and triacetylated (A_3_) oligomers.

## References

[1] Medzhitov R. (2008). Origin and physiological roles of inflammation. Nature.

[2] Kawai T., Akira S. (2010). The role of pattern-recognition receptors in innate immunity: Update on Toll-like receptors. Nat. Immunol..

[3] Abreu M.T., Thomas L.S., Arnold E.T., CLukasek K., Michelsen K.S., Arditi M. (2003). TLR signaling at the intestinal epithelial interface. J. Endotoxin Res..

[4] Arndt P.G., Suzuki N., Avdi N.J., Malcolm K.C., Worthen G.S. (2004). Lipopolysaccharide-induced c-Jun NH2-terminal kinase activation in human neutrophils: Role of phosphatidylinositol 3-Kinase and Syk-mediated pathways. J. Boil. Chem..

[5] Kolaczkowska E., Kubes P. (2013). Neutrophil recruitment and function in health and inflammation. Nat. Rev. Immunol..

[6] Perobelli S.M., Galvani R.G., Gonçalves-Silva T., Xavier C.R., Nóbrega A., Bonomo A. (2015). Plasticity of neutrophils reveals modulatory capacity. Braz. J. Med Boil. Res..

[7] Sica A., Mantovani A. (2012). Macrophage plasticity and polarization: In vivo veritas. J. Clin. Investig..

[8] Stout R.D., Suttles J. (2004). Functional plasticity of macrophages: Reversible adaptation to changing microenvironments. J. Leukocite Boil..

[9] Johnson G.L., Lapadat R. (2002). Mitogen-activated protein kinase pathways mediated by ERK, JNK, and p38 protein kinases. Science.

[10] Manning G., Whyte D.B., Martinez R., Hunter T. (2002). Sudarsanam, S. The protein kinase complement of the human genome. Science.

[11] Sabio G., Davis R.J. (2014). TNF and MAP kinase signalling pathways. Semin. Immunol..

[12] English J.M., Cobb M.H. (2002). Pharmacological inhibitors of MAPK pathways. Trends Pharmacol. Sci..

[13] Li K., Xing R., Liu S., Li P. (2016). Advances in preparation, analysis and biological activities of single chitooligosaccharides. Carbohydr. Polym..

[14] Ngo D.H., Vo T.S., Ngo D.N., Kang K.H., Je J.Y., Pham N.D., Byun H.G., Kim S.K. (2015). Biological effects of chitosan and its derivatives. Food Hydrocoll..

[15] Xu Q., Liu M., Liu Q., Wang W., Du Y., Yin H. (2017). The inhibition of LPS-induced inflammation in RAW264.7 macrophages via the PI3K/Akt pathway by highly N-acetylated chitooligosaccharide. Carbohydr. Polym..

[16] Zou P., Yang X., Wang J., Li Y., Yu H., Zhang Y., Liu G. (2016). Advances in characterization and biological activities of chitosan and chitosan oligosaccharides. Food Chem..

[17] Santos-Moriano P., Woodley J.M., Plou F.J. (2016). Continuous production of chitooligosaccharides by an immobilized enzyme in a dual-reactor system. J. Mol. Catal. B Enzym..

[18] Chen M., Zhu X., Li Z., Guo X., Ling P. (2010). Application of matrix-assisted laser desorption/ionization time-of-flight mass spectrometry (MALDI-TOF-MS) in preparation of chitosan oligosaccharides (COS) with degree of polymerization (DP) 5–12 containing well-distributed acetyl groups. Int. J. Mass Spectrom..

[19] Tsao C.T., Chang C.H., Lin Y.Y., Wu M.F., Han J.L., Hsieh K.H. (2011). Kinetic study of acid depolymerization of chitosan and effects of low molecular weight chitosan on erythrocyte rouleaux formation. Carbohydr. Res..

[20] Wu S. (2011). Preparation of water soluble chitosan by hydrolysis with commercial a-amylase containing chitosanase activity. Food Chem..

[21] Xia Z., Wu S., Chen J. (2013). Preparation of water soluble chitosan by hydrolysis using hydrogen peroxide. Int. J. Boil. Macromol..

[22] Xing R., Liu Y., Li K., Yu H., Liu S., Yang Y., Chen X., Li P. (2017). Monomer composition of chitooligosaccharides obtained by different degradation methods and their effects on immunomodulatory activities. Carbohydr. Polym..

[23] Cabrera J.C.P., Cutsem P. (2005). Preparation of chitooligosaccharides with degree of polymerization higher than 6 by acid or enzymatic degradation of chitosan. Biochem. Eng. J..

[24] Hamer S.N., Cord-Landwehr S., Biarnés X., Planas A., Waegeman H., Moerschbacher B.M., Kolkenbrock S. (2015). Enzymatic production of defined chitosan oligomers with a specific pattern of acetylation using a combination of chitin oligosaccharide deacetylases. Sci. Rep..

[25] Jung W.J., Park R.D. (2014). Bioproduction of Chitooligosaccharides: Present and Perspectives. Mar. Drugs.

[26] Vårum K.M., Holme H.K., Izume M., Torger-Stokke B., Smidsrød O. (1996). Determination of enzymatic hydrolysis specificity of partially N-acetylated chitosans. Biochim. Biophys. Acta.

[27] Aam B.B., Heggset E.B., Norberg A.L., Sørlie M., Vårum K.M., Eijsink V.G.H. (2010). Production of Chitooligosaccharides and Their Potential Applications in Medicine. Mar. Drugs.

[28] Weinhold M.X., Sauvageau J.C.M., Kumirska J., Thöming J. (2009). Studies on acetylation patterns of different chitosan preparations. Carbohydr. Polym..

[29] Tømmeraas K., Vårum K.M., Christensen B.E., Smidsrød O. (2001). Preparation and characterization of oligosaccharides produced by nitrous acid depolymerisation of chitosans. Carbohydr. Res..

[30] Soria-Castro I., Krzyzanowska A., Pelaez M.L., Regadera J., Ferrer G., Montoliu L., Rodriguez-Ramos R., Fernandez M., Alemany S. (2010). Cot/tpl2 (MAP3K8) mediates myeloperoxidase activity and hypernociception following peripheral inflammation. J. Boil. Chem..

[31] Cardoso L.S., Araujo M.I., Góes A.M., Pacífico L.G., Oliveira R.R., Oliveira S.C. (2007). Polymyxin B as inhibitor of LPS contamination of Schistosoma mansoni recombinant proteins in human cytokine analysis. Microb. Cell Fact..

[32] MacLean-Fletcher S., Pollard T.D. (1980). Mechanism of action of cytochalasin B on actin. Cell.

[33] Ishiguro K., Yoshie N., Sakurai M., Inoue Y. (1992). A 1H-NMR study of a fragment of partially N-deacetylated chitin produced by lysozyme degradation. Carbohydr. Res..

[34] Jung W.K., Park P.J., Ahn C.B., Je J.Y. (2014). Preparation and antioxidant potential of maillard reaction products from (MRPs) chitooligomer. Food Chem..

[35] Mengíbar M., Ganan M., Miralles B., Carrascosa A.V., Martínez-Rodriguez A.J., Peter M.G., Heras A. (2011). Antibacterial activity of products of depolymerization of chitosans with lysozyme and chitosanase against Campylobacter jejuni. Carbohydr. Polym..

[36] Sánchez Á., Mengíbar M., Rivera-Rodríguez G., Moerchbacher B., Acosta N., Heras A. (2017). The effect of preparation processes on the physicochemical characteristics and antibacterial activity of chitooligosaccharides. Carbohydr. Polym..

[37] Fernandes J.C., Spindola H., de Sousa V., Santos-Silva A., Pintado M.E., Malcata F.X., Carvalho J.E. (2010). Anti-Inflammatory Activity of Chitooligosaccharides in vivo. Mar. Drugs.

[38] Qiao Y., Bai X., Du Y. (2011). Chitosan oligosaccharides protect mice from LPS challenge by attenuation of inflammation and oxidative stress. Int. Immunopharmacol..

[39] Yoon H.J., Moon M.E., Park H.S., Im S.Y., Kim Y.H. (2007). Chitosan oligosaccharide (COS) inhibits LPS-induced inflammatory effects in RAW 264.7 macrophage cells. Biochem. Biophys. Res. Commun..

[40] Davydova V.N., Yermak I.M., Gorbach V.I., Krasikova I.N., Solov’eva T.F. (2000). Interaction of Bacterial Endotoxins with Chitosan. Effect of Endotoxin Structure, Chitosan Molecular Mass, and Ionic Strength of the Solution on the Formation of the Complex. Biochemistry.

[41] Yermak I.M., Davidova V.N., Gorbach V.I., Luk’yanov P.A., Solov’eva T.F., Ulmer A.J., Buwitt-Beckmann U., Rietschel E.T., Ovodov Y.S. (2006). Forming and immunological properties of some lipopolysaccharide–chitosan complexes. Biochimie.

[42] Davydova V.N., Naberezhnykh G.A., Yermak I.M., Gorbach I.N., Solov’eva T.F. (2006). Determination of Binding Constants of Lipopolysaccharides of Different Structure with Chitosan. Biochemistry.

[43] Naberezhnykh G.A., Gorbach V.I., Likhatskaya G.N., Davidova V.N., Solov’eva T.F. (2008). Interaction of Chitosans and Their N-Acylated Derivatives with Lipopolysaccharide of Gram-Negative Bacteria. Biochemistry.

[44] Naberezhnykh G.A., Gorbach V.I., Kalmykova E.N., Solov’eva T.F. (2015). Determination of the parameters of binding between lipopolysaccharide and chitosan and its N-acetylated derivative using a gravimetric piezoquartz biosensor. Biophys. Chem..

[45] Cagnol S., Chambard J.C. (2010). ERK and cell death: Mechanisms of ERK-induced cell death--apoptosis, autophagy and senescence. FEBS J..

[46] Khavari T.A., Rinn J. (2007). Ras/Erk MAPK signaling in epidermal homeostasis and neoplasia. Cell Cycle.

[47] Mebratu Y., Tesfaigzi Y. (2009). How ERK1/2 activation controls cell proliferation and cell death: Is subcellular localization the answer?. Cell Cycle.

[48] Minakami M., Kitagawa N., Iida H., Anan H., Inai T. (2015). p38 mitogen-activated protein kinase and c-jun NH2-terminal protein kinase regulate the accumulation of a tight junction protein, ZO-1, in cell–cell contacts in HaCaT cells. Tissue Cell.

[49] Capuano E., Fogliano V. (2011). Acrylamide and 5-hydroxymethylfurfural (HMF): A review on metabolism, toxicity, occurrence in food and mitigation strategies. LWT–Food Sci. Technol..

[50] NTP (2010). NTP toxicology and carcinogenesis studies of 5-(Hydroxymethyl)-2-furfural (CAS No. 67-47-0) in F344/N rats and B6C3F1 mice (gavage studies). Natl. Toxicol. Program Tech. Rep. Ser..

[51] Tomas D., Karin K., Stephanie D., Imre B. (2005). Analysis of Amadori compounds by high-performance cation exchange chromatography coupled to tandem mass spectrometry. Anal. Chem..

[52] Zeng L., Qin C., Chi W., Wang L., Ku Z., Li W. (2007). Browning of chitooligomers and their optimum preservation. Carbohydr. Polym..

[53] Basta G. (2008). Receptor for advanced glycation end products and atherosclerosis: From basic mechanisms to clinical implications. Atherosclerosis.

[54] Akira S., Uematsu S., Takeuchi O. (2006). Pathogen recognition and innate immunity. Cell.

[55] Ott C., Jacobs K., Haucke E., Navarrete Santos A., Grune T., Simm A. (2014). Role of advanced glycation end products in cellular signaling. Redox Boil..

[56] Allan G.G., Peyron M. (1995). Molecular weight manipulation of chitosan I: Kinetics of depolymerization by nitrous acid. Carbohydr. Res..

[57] Mengíbar M., Mateos-Aparicio I., Miralles B., Heras A. (2013). Influence of the physico-chemical characteristics of chito-oligosaccharides (COS) on antioxidant activity. Carbohydr. Polym..

[58] Hirai A., Odani H., Nakajima A. (1991). Determination of degree of deacetylation of chitosan by 1H- NMR spectroscopy. Polym. Bullettin.

[59] Madhuprakash J., El Gueddari N.E., Moerchbacher B.M., Podile A.R. (2015). Production of bioactive chitosan oligosaccharides using the hypertransglycosylating chitinase-D from serratia proteamaculans. Bioresour. Technol..

[60] Hansen M.B., Nielsen S.E., Berg K. (1989). Re-examination and further development of a precise and rapid dye method for measuring cell growth/cell kill. J. Immunol. Methods.

[61] Rodríguez C., López P., Pozo M., Duce A.M., López-Pelaéz M., Fernández M., Alemany S. (2008). COX2 expression and Erk1/Erk2 activity mediate Cot-induced cell migration. Cell. Signal.

